# Efficiency of Crizotinib on an ALK-Positive Inflammatory Myofibroblastic Tumor of the Central Nervous System: A Case Report

**DOI:** 10.7759/cureus.1068

**Published:** 2017-03-02

**Authors:** Anas Chennouf, Elizabeth Arslanian, David Roberge, France Berthelet, Michel Bojanowski, Jean-Paul Bahary, Laura Masucci, Karl Belanger, Marie Florescu, Philip Wong

**Affiliations:** 1 CRCHUM, Université de Montréal; 2 Pathology, Centre Hospitalier de l'Université de Montréal (CHUM); 3 Department of Oncology, Division of Radiation Oncology, McGill University Health Center; 4 Neurosurgery, Centre Hospitalier de l'Université de Montréal (CHUM); 5 Radiation Oncology, Centre hospitalier de l'université de Montréal (CHUM) - Hôpital Notre-Dame; 6 Department of Radiation Oncology, Centre Hospitalier de l'Université de Montréal (CHUM); 7 Hemato-Oncology, Centre Hospitalier de l'Université de Montréal (CHUM)

**Keywords:** inflammatory myofibroblastic tumor, crizotinib, alk, radiotherapy, cns, response

## Abstract

Inflammatory myofibroblastic tumors (IMT) of the central nervous system (CNS) are rare entities that have a predilection for local recurrences. Approximately half of the inflammatory myofibroblastic tumors contain translocations that result in the over-expression of the anaplastic lymphoma kinase (ALK) gene. We hereby present the case of a patient diagnosed with a left parieto-occipital IMT that recurred after multiple surgeries and radiotherapy. Immuno-histochemical examination of the tumor demonstrated ALK overexpression and the presence of an ALK rearrangement observed in lung cancers. The patient was subsequently started on an ALK inhibitor. A response evaluation criteria in solid tumors (RECIST) partial response was observed by the seventh month of ALK inhibition and the tumor remained in control for 14 months. The current case reiterates the activity of ALK inhibitors within the CNS and suggests that radiotherapy may potentiate the permeability of ALK inhibitors in CNS tumors addicted to ALK signalling.

## Introduction

Inflammatory myofibroblastic tumors (IMTs) are neoplastic lesions that are typically benign but may rarely (< five percent) metastasize. Histopathologically, these lesions consist of myofibroblastic spindle cells within an inflammatory environment consisting of lymphocytes, plasma cells, eosinophils, histiocytes, and abundant blood vessels. Published reports of IMTs suggest that this disease often originates in the viscera and soft-tissues of children and young adults, with the mean age of diagnosis being 13.2 years old [1]. In the central nervous system (CNS), IMTs tend to arise from meningeal structures and have a higher potential of recurrence and malignant transformation than non CNS-IMTs. A few reported cases described a higher recurrence rate in IMTs overexpressing the anaplastic lymphoma kinase (ALK) protein in these specific tumors [2], resulting in complex clinical situations.

We present the case of a patient with an ALK positive cerebellar IMT treated with an ALK inhibitor (crizotinib) after multiple local recurrences following four surgical resections and radiotherapy. Informed consent was obtained from the patient for this study.

## Case presentation

A 26-year-old male presented to the emergency with symptoms of left hemi-cranial headaches that exacerbated overnight, bilateral scintillating scotoma, and slight gait imbalances in November 2010. The neurological examination was within the normal limits except for a right homonymous hemianopia. The patient’s history was significant for asthma and a 10 pack-year smoking history. There was no recent infection or trauma. A contrast-enhanced computed tomography (CT) scan showed the presence of a left parieto-occipital 4.8 x 4.7 x 4.9 cm mass with a slight hypodense area in the petrous apex. On MRI, the lesion was gadolinium-enhanced and originated from the tentorium cerebelli, along with local vasogenic oedema and a 5 mm right midline deviation. Angiography suggested that the tumor was poorly vascularized. Blood work was normal. The initial differential diagnosis consisted of glioma, lymphoma, tentorial meningioma and solitary fibrous tumor/hemangiopericytoma.

**Figure 1:**
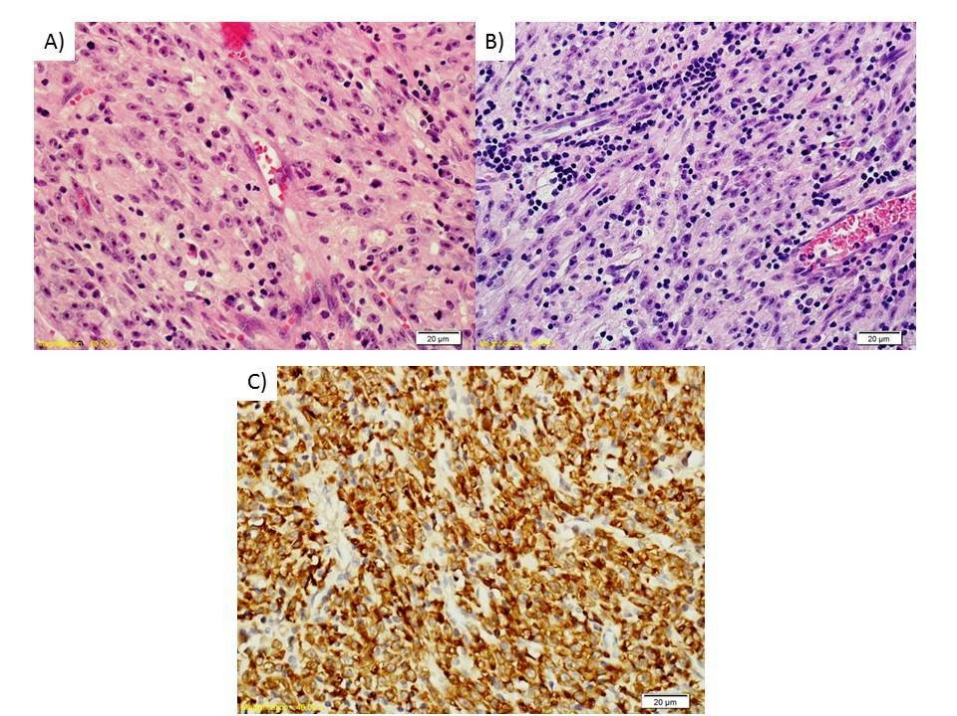
Histological images of the tumor Histologically, the tumors excised from the first surgery (A) and at the last surgery (B) represent very typical findings of an inflammatory myofibroblastic tumor. The tumor is comprised of plump spindle cells in a collagenized background and abundant blood vessels. There is a distinctive inflammatory infiltrate with small aggregates of lymphocytes and plasma cells. The cells contained large nuclei and nucleoli. Strong cytoplasmic staining for ALK was noted (C). There was an average of four mitoses per 10 high-power fields (HPF).

Ten days later, the patient underwent a partial resection (A) of the mass through a left parieto-occipital craniotomy. Following a pathology review, the tumor was diagnosed as an inflammatory myofibroblastic tumor (Figure 1). Post-intervention MRI performed the next day showed that a residual 3.3 x 1.5 cm tumor lesion was still present along with normal postoperative modifications. No suspicious distant lesions were found on a positron emission tomography-computed tomography (PET-CT) performed two months following the surgery.

### Progression and treatment

**Figure 2:**
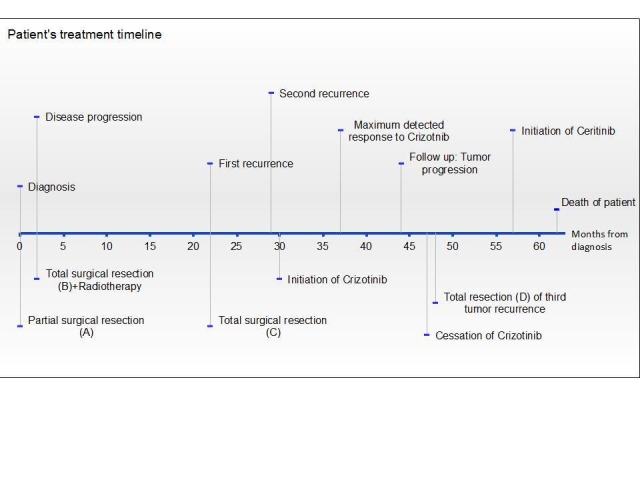
Timeline of patient’s diagnosis and treatments

The patient’s timeline from the time of diagnosis is presented in Figure 2. In February 2011, two months after the operation (A), the patient started having symptoms of important headaches, behaviour changes, and aphasia. A follow-up MRI showed a 4.4 x 3.8 x 3.0 cm left temporo-parietal mass suggesting a progression from the residual tumor.

A gross total resection (B) of the tumor was completed a week later and no residual lesion was found on postoperative head CT and MRI performed one and two days later, respectively. Pathological examination again confirmed an IMT with histological findings similar to the original resection (A). To reduce the risk of local recurrence, the patient was given adjuvant radiotherapy consisting of 60 Gy in 30 fractions to the postoperative tumor bed plus a 1 cm planning target volume (PTV) margin. Radiotherapy was well tolerated by the patient except for intermittent diplopia that resolved few weeks after the completion of the treatments. A collection of cerebral spinal fluid was also found in his middle ear, which indicated the presence of a fistula of the left mastoid. This was repaired surgically a week later. The patient was followed using MRIs at three, six, and 10 months post-resection (B) and had no sign of residual disease or recurrence.

Other recurrences and treatment with ALK inhibitors

     1) First recurrence and operation outcome 2) Second recurrence and treatment with crizotinib 3) Tumor follow-up and crizotinib’s effect 4) Third recurrence and treatment with ceritinib

(1) The patient remained well and asymptomatic until twenty months post-op (B) in October 2012 when he started developing repetitive inappropriate laughter, aggressiveness, and transient aphasia. A head CT-scan revealed the presence of a 4.7 x 3.6 x 3.0 cm local recurrence extending to the tentorial incisure and compressing the left temporal lobe and superior cerebellum. A concomitant craniotomy and mastoidectomy (C) was performed five days later and the tumor was excised. A CT-scan at 10 days post-op showed no residual disease but noted the presence of a postoperative pseudomeningocele. Pathology review once again confirmed an IMT recurrence. A follow-up MRI at three months post-op showed no residual tumor.

(2) In April 2013, a follow-up CT scan revealed the presence of a 1.4 x 1.1 cm left tentorial nodule suspicious of a recurrence. Since the patient had already undergone four extensive surgeries in the last two years, radiosurgery was proposed to him instead of surgery. However, a CT scan performed a month later indicated a progression of the lesion (1.7 x 1.3 cm) and the appearance of two new 0.8 x 0.8 cm supra-tentorial nodules.

Given the rapid evolution and limited treatment alternatives, the patient’s case was reviewed at the neuro-oncology multi-disciplinary tumor board. An evaluation for ALK rearrangement was requested and performed using the Vysis LSI ALK Break Apart Rearrangement FISH Probe Kit (Abbott Molecular, IL, USA). Of the 52 cells evaluated, 31 (60%) had an ALK rearrangement. Through extrapolation of lung cancer data suggesting a high response rate to the ALK inhibitor [3] among ALK-positive cancers, the patient was offered a trial course of crizotinib at a dose of 250 mg twice a day (BID) started in May 2013. This treatment was subsequently maintained.

(3) In July 2013, two months following the initiation of crizotinib, a reduction in the size of the primary nodule (from 1.7 x 1.3 cm to 1.3 x 0.7 cm) and disappearance of the two other 0.8 x 0.8 cm supra-tentorial nodules was observed on MRI. There were no new enhancing foci detected.

Meanwhile, the patient had several electrocardiograms and cardiology follow-ups due to asymptomatic QT prolongation, a known side effect of crizotinib. Magnesium was prescribed to avoid potential *torsade de pointes* and was later stopped when the QT prolongation was resolved. During these appointments, the patient complained of insomnia, dysgeusia and diarrhea, and had a slight tremor during the finger to nose test. He also described having visual disturbances that had resolved after a two- to three-month period.

**Figure 3:**
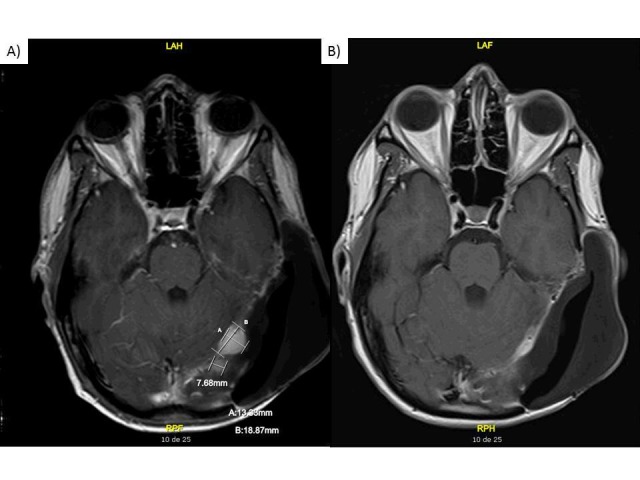
MRI images while the patient was on crizotinib Axial T1 post-contrast (gadolinium) MRI from A) initiation of crizotinib and B) seven months later.

In December 2013, MRI showed further reduction in the size of the tentorial nodule (0.9 x 0.8 cm), thus representing a 52% size reduction and a response evaluation criteria in solid tumors (RECIST) V1.1 partial response in comparison to the pre-crizotinib MRI (Figures 3A-3B). Physical exam was normal and the patient had no symptoms except for a persistent diarrhea treated with Imodium.

A gadolinium-enhanced MRI completed three months later revealed a small focus near the cerebellar tentorium. Subsequent imaging revealed slow millimetric growth of the enhancing lesion. As the patient remained asymptomatic, he was maintained on crizotinib.

(4) In August 2014, 14 months after the start of crizotinib, the patient presented with a clinical deterioration of his condition. He was having important headaches, mood swings, memory loss, and paresthesias in his upper right member that had started three weeks before his scheduled visit. MRI showed that the lesion had grown to 3.4 x 1.9 cm and was extending to both sides of the cerebellar tentorium. Crizotinib was stopped in September 2014. He underwent a fourth resection (D) and enrolled into a special access program to receive ceritinib (a 2^nd^ generation ALK inhibitor) nine months post-surgery. However, the patient refused to continue receiving ceritinib soon after program initiation. He passed away five months later, in January 2016.

## Discussion

IMTs are rare tumors exhibiting various histological features, aggressiveness patterns and symptomatology. Currently, there is no clear-cut treatment algorithm for IMTs, although macroscopic surgical excision remains the treatment of choice [4]. The use of radiotherapy, corticosteroids, and chemotherapy has also been described in the literature for recurrent or unresectable tumors. More recently, the use of ALK inhibitors such as crizotinib and ceritinib in the treatment of IMTs has been added to the National Comprehensive Cancer Network (NCCN) guidelines for metastatic disease.

Recent developments in genetics and targeted therapies increased the number of molecular tools to aid pathologists in refining their diagnosis and oncologists in selecting agents targeting specific molecular alterations in a particular tumor. In the case of IMTs, approximately 50% of CNS-IMTs are known to harbour a cytogenetic translocation that upregulates the ALK gene located at 2p23 locus resulting in overexpression of ALK protein. The pathogenesis of the remaining half of IMTs lacking ALK expression is not clear.

ALK inhibitors have been used in several clinical trials for ALK-positive tumor treatments. Crizotinib is the first ALK tyrosine kinase inhibitor to become clinically available and has shown marked and durable efficacy for the treatment of non-small cell lung cancer (NSCLC) positive for ALK rearrangement [5]. However, crizotinib is considered to have limited efficacy in brain metastases and primary CNS tumors due to its limited penetration across the blood brain barrier (BBB). Ross Camidge reported on seven cases of ALK-positive NSCLC that responded to crizotinib outside the CNS, but progressed within the CNS [6]. However, the combination of radiotherapy with an ALK inhibitor may improve the efficacy of ALK inhibitors as radiotherapy increases the permeability of the BBB, thereby improving the drug’s penetration into the CNS. Two prior case reports on NSCLC [5] and glioblastoma [7] support the potential of this drug-radiotherapy combination. Using dynamic contrast-enhanced MRIs, previous studies had previously observed a continued increase in BBB permeability at six months post-radiotherapy (20-60 Gy), which correlated with long-term neurocognitive dysfunctions of patients [8]. Other studies similarly observed permeability changes in normal brain parenchyma two to ten years following radiotherapy [9]. In this case, although the patient received radiotherapy 25 months prior to crizotinib, the effect of radiotherapy and prior surgeries on the permeability of the BBB may still be present.

To our knowledge, this is the first report of an ALK-positive CNS-IMT that has resulted in a RECIST defined partial response to crizotinib. This response and duration of tumor control may have been potentiated by the prior course of brain radiotherapy that permeabilized the BBB and/or tumor vasculature. Resistance to crizotinib generally occurs in patients within one to two years [10]. The patient presented in this case had a 10-month period of tumor regression followed by a four-month period of slow tumor regrowth, which may be secondary to the clonal expansion of a tumor subpopulation. As the patient chose not to pursue further ceritinib therapy, we cannot deduce whether ceritinib could overcome crizotinib resistance as previously observed in NSCLC.

## Conclusions

Inflammatory myofibroblastic tumors occurring in the central nervous system have a high tendency to recur. Initial treatment with complete surgical resection remains the primary treatment. At the time of recurrence, the tumor sample should be tested for ALK rearrangement, as ALK inhibitors may be an efficacious treatment against ALK-rearranged IMTs. In CNS-IMTs, sequential or concurrent radiotherapy may represent a strategy to improve the activity of ALK inhibitors in the brain.
